# Validation of a core outcome measure for palliative care in Africa: the APCA African Palliative Outcome Scale

**DOI:** 10.1186/1477-7525-8-10

**Published:** 2010-01-25

**Authors:** Richard Harding, Lucy Selman, Godfrey Agupio, Natalya Dinat, Julia Downing, Liz Gwyther, Thandi Mashao, Keletso Mmoledi, Tony Moll, Lydia Mpanga Sebuyira, Barbara Panjatovic, Irene J Higginson

**Affiliations:** 1 King's College London, Dept Palliative Care, Policy & Rehabilitation, King's College London, Weston Education Centre, Cutcombe Road, Denmark Hill, London SE5 9RJ, UK; 2 Hospice Africa Uganda, Plot 130 Makindye Road, PO Box 7757, Kampala, Uganda; 3 Witwatersrand Palliative Care, PO Box 212, Pimville, Soweto 1808, Johannesburg, Gauteng, South Africa; 4 African Palliative Care Association, PO Box 72518, Kampala, Uganda; 5 Hospice Palliative Care Association of South Africa, PO Box 38785, Pinelands 7430, Cape Town, Western Cape, South Africa; 6 Palliative Medicine Unit, University of Cape Town, Anzio Road, Observatory 7925, Cape Town, Western Cape, South Africa; 7 Church of Scotland Hospital, P/Bag X502, Tugela Ferry 3010, KwaZulu Natal, South Africa; 8 Infectious Diseases Institute, Faculty of Medicine, Makerere University, PO Box 22418, Kampala, Uganda; 9 Msunduzi Hospice, PO Box 220223, Mayor's Walk, Pietermaritzburg 3208, KwaZulu Natal, South Africa

## Abstract

**Background:**

Despite the burden of progressive incurable disease in Africa, there is almost no evidence on patient care or outcomes. A primary reason has been the lack of appropriate locally-validated outcome tools. This study aimed to validate a multidimensional scale (the APCA African Palliative Outcome Scale) in a multi-centred international study.

**Methods:**

Validation was conducted across 5 African services and in 3 phases: Phase 1. Face validity: content analysis of qualitative interviews and cognitive interviewing of POS; Phase 2. Construct validity: correlation of POS with Missoula-Vitas Quality of Life Index (Spearman's rank tests); Phase 3. Internal consistency (Cronbach's alpha calculated twice using 2 datasets), test-retest reliability (intraclass correlation coefficients calculated for 2 time points) and time to complete (calculated twice using 2 datasets).

**Results:**

The validation involved 682 patients and 437 family carers, interviewed in 8 different languages. Phase 1. Qualitative interviews (N = 90 patients; N = 38 carers) showed POS items mapped well onto identified needs; cognitive interviews (N = 73 patients; N = 29 carers) demonstrated good interpretation; Phase 2. POS-MVQoLI Spearman's rank correlations were low-moderate as expected (N = 285); Phase 3. (N = 307, 2nd assessment mean 21.2 hours after first, SD 7.2) Cronbach's Alpha was 0.6 on both datasets, indicating expected moderate internal consistency; test-retest found high intra-class correlation coefficients for all items (0.78-0.89); median time to complete 7 mins, reducing to 5 mins at second visit.

**Conclusions:**

The APCA African POS has sound psychometric properties, is well comprehended and brief to use. Application of this tool offers the opportunity to at last address the omissions of palliative care research in Africa.

## Background

The lack of clinical and research activity to enhance care of the dying among those HIV-infected is a global challenge. Despite two million deaths during 2007, with emerging international data reporting high mortality even as access to therapy increases, very little scientific attention is paid to improving the experience of death and dying [1].

The burden of progressive, life-limiting disease in Sub-Saharan Africa is reflected in the epidemiology of HIV [2,3] and cancer [4]. In sub-Saharan Africa during 2007 there were 22.5 million people living with HIV infection; 1.7 million adults and children became infected with HIV; and 1.6 million died of AIDS [1]. Based on GLOBOCAN 2002 cancer rates and UN population predictions, there were an estimated 7.6 million new cancer cases and 6 million deaths from cancer in Africa in 2007 [5], and malignancies are a common presentation of HIV progression. The burden of other progressive non-malignant diseases is unknown.

Significant advances have been achieved in African palliative care provision to manage the highly prevalent and burdensome problems experienced by those with incurable terminal disease. However, there is very little evidence for outcomes of effectiveness of this care, a common problem in developing country contexts, where health systems research is under-funded [6,7]. A primary reason for this dearth of evidence is the lack of appropriate and validated outcome tools [8], among other logistical and methodological challenges in this setting and population [9].

Advanced care clinicians in Africa identified the need for appropriate outcome tools [10], and suggested that these tools should be appropriate for both HIV and cancer patients, address family and patient outcomes, be locally validated [11], and be relevant to all stages of the disease trajectory [12]. In addition to validity and reliability, key principles of outcome tools are brevity and multidimensionality, i.e. addressing physical, emotional, spiritual and social problems of both patients and families. Self-completion tools are often inappropriate for patients with advanced illness, and may not be feasible in populations with limited literacy.

To date, only one palliative measure has been validated in Africa [13]. The Missoula Vitas Quality of Life Index (MVQoLI) is a 25-item measure developed in the USA and validated in Uganda. However, the tool was originally designed for use in clinical care, and was not studied comprehensively as an outcome measure. Psychometric testing of a revised version of the tool concluded that it does not have appropriate properties for outcomes research in patients with advanced illness [14].

The original Palliative Outcome Scale (POS) is a 10-item multidimensional tool [15], adapted and validated globally in a number of cultural and linguistic versions [16-18]. Independent assessments of the utility of the POS have identified it as useful and valid in clinical audit, training and research [19,20]. This study reports the validation (i.e. investigation of the degree to which the instrument accurately and reliably measures what it intends to) of the APCA African POS, a tool developed by a multi-professional expert panel and piloted in 11 sites in 8 Eastern and Southern African countries (Botswana, Kenya, Malawi, South Africa, Tanzania, Uganda, Zambia and Zimbabwe). The developmental pre-clinical phase has been reported previously (i.e. content and consensus validity) [21], and tested whether the measure could: (a) yield information of clinical relevance to palliative care, (b) cover those domains considered to be important to this type of care and nothing more, and (c) achieve a consensus among specialists that (a) and (b) had been met. Subsequent consultation was undertaken with a panel of African clinicians [11]. During this developmental phase, sensitivity to change was also reported on the original pool of potential items.

In this paper we report the full, international, multi-centre clinical validation of the tool.

## Methods

**Figure 1:**
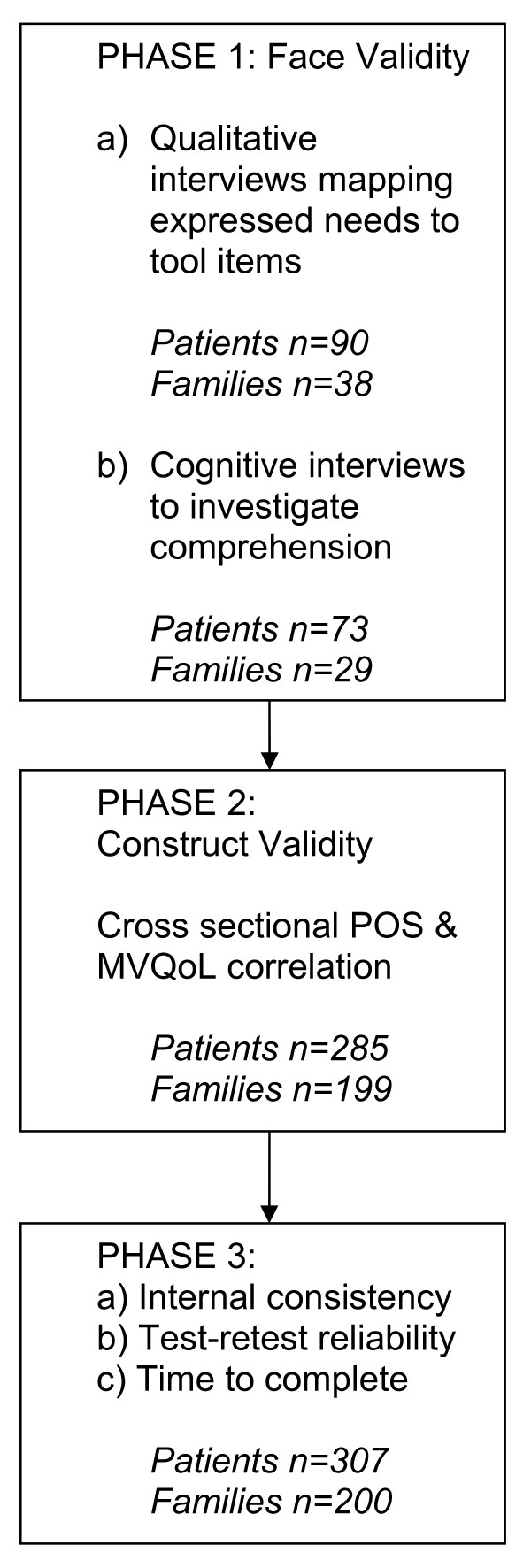
**Study Design**.

This validation study used a 3-phase clinical study design: Phase 1 Face validity; Phase 2 Construct validity; Phase 3 Internal consistency, test/re-test reliability and time to complete (Figure 1).

### Participating Sites

The validation was undertaken in 5 palliative care sites, 4 in South Africa and 1 in Uganda, based in rural, peri-urban and urban areas, including homecare, day care and inpatient facilities. Two of the sites provide care from the point of diagnosis through to the end of life while the remaining 3 focus primarily on advanced disease.

### Recruitment

Inclusion criteria were adult patients (at least 18 years old) under care with sufficient physical and cognitive ability to participate in interviews. All information and consent forms and tools were translated from English (forward and back) into the principle languages of Luganda, Runyankole, Sesotho, Runyoro, SeTswana, isiXhosa and 2 isiZulu dialects. Informed consent was obtained from all participants. The study was reviewed and approved by the Ethical Review Boards of the Universities of Cape Town, KwaZulu Natal and Witwatersrand, the Ugandan National Council for Science and Technology, Hospice Africa Uganda and the Hospice Palliative Care Association of South Africa.

### The APCA African POS

The APCA African POS contains 10 items, addressing the physical and psychological symptoms, spiritual, practical and emotional concerns, and psychosocial needs of the patient and family (see Additional File 1). The answers to all questions are scored using Likert scales from 0 to 5, with numerical and descriptive labels. Questions 1-7 are directed at patients; questions 8-10 are directed at family informal caregivers and include a 'Not applicable' option for use when the patient does not have an informal carer. The African version of the POS is staff-completed, owing to varying levels of patient and family literacy. Respondents indicate their answers either verbally or using a hand scale (0 = closed fist, 5 = all fingers open). The responses use a combination of high score = best status and low score = best status as a mechanism to ensure that administration, and response formulation to the individual items, are conducted with due care and attention. The tool used throughout this validation study was not changed from the original development study.

### Validity and Reliability

The measure was tested by evaluating the components of validity and reliability described below. Trained palliative care research nurses (TM, KM, GA and two others) and three assistants administered all testing procedures.

#### i. Face validity: patients' and carers' views (Phases 1a and 1b)

Face validity relates to the appropriateness and acceptability of the measure to the target population. In-depth qualitative interviews with patients and carers were conducted in order to ensure that domains of need mapped on to POS items (Phase 1a). Cognitive interviews were conducted to explore whether the respondents found any questions confusing, upsetting, or irrelevant, to understand perceived meaning, to determine how they formulated responses, and identify whether any important issues were felt to be missing (Phase 1b) [22].

#### ii. Construct validity (Phase 2)

Assessing construct validity ideally involves comparing a measure with a different measure of the same construct that has previously been validated in the same population, in order to determine convergence or divergence. The only palliative care scale previously validated in a similar population was the MVQoLI [13]. The original MVQoLI is a measure of quality of life during advanced illness. Its distinctive features are a scoring system that allows the weighting of each dimension of QOL by the respondent, and the subjective wording of the items that allows respondents to interpret the measured elements according to their own experience [23]. Patients completed both the POS and MVQoLI at a single time point. POS carer items (8-10) were completed by family carers where available. The MVQoLI is divided into 5 subscales: symptoms, function, well being, interpersonal and transcendent. There are important differences between the two measures. The MVQoLI is considerably longer than the POS (26 items compared to 10), the MVQoLI does not measure family carers' well being, and the MVQoLI addresses physical function. Owing to these differences, it was hypothesised that a high degree of correlation would not be found (i.e. that correlation would be less than 0.6).

#### iii. Internal consistency (Phase 3)

Testing for internal consistency involves estimating how consistently individuals respond to the items within a scale. Where items within a scale measure different elements of patient experience (as in this multidimensional tool) a moderate Cronbach's alpha (i.e. approximately 0.5), rather than a high alpha (i.e. >0.7), is expected.

#### iv. Test/re-test reliability (Phase 3)

Test/retest reliability measures the stability of a measure over a short time period, i.e. determines whether a measure is sensitive to change but not so sensitive as to report clinically insignificant changes. Test/re-test reliability was measured on two consecutive visits within 5-48 hours.

#### v. Time to complete (Phase 3)

Time taken to complete a measure is important when assessing appropriateness for a patient group and use in clinical practice, particularly in populations living with advanced illness. Research nurses timed the administration of the POS during the test/re-test reliability phase to gauge time to complete the tool under typical repeated use. During Phase 2, time to complete the MVQoLI was also recorded for comparison purposes.

### Demographic data

For each phase of the validation, the following patient demographic and clinical data were collected: age, gender, first language, language of interview, diagnosis (for cancer patients, also cancer type; for HIV patients, also antiretroviral (ART) treatment status, prior AIDS diagnosis), household size, number of children responsible for, location of home (urban, peri-urban, rural), primary place of care (home, inpatient/outpatient unit, day care facility), functional status (using the ECOG [24]), and time under care in weeks. In the qualitative phase of validation family carers reported age, gender, first language, language of interview, household size, number of children responsible for, and location of home (urban, peri-urban, rural).

We elected to collect data on the number of children that respondents were responsible for, rather than number of biological children. This was because within Africa multiple AIDS deaths within the same family, and broader concepts of what constitutes "family", mean adults may care for children other than their own, e.g. grandchildren, nephews and nieces.

### Translation and data capture

The APCA African POS, MVQoLI, qualitative interview schedule, demographic record, and information and consent sheets were translated from English into the main local languages (isiXhosa, isiZulu (Gauteng and KwaZulu Natal dialects), SeSotho, SeTswana, Luganda and Runyoro). The translations were undertaken in Academic departments hosting the research and were therefore professional in their skills and knowledge of both language and topic. The research nurses entered quantitative data into purpose-designed Excel spreadsheets, subsequently imported into SPSS for analysis. Qualitative and cognitive interviews were conducted in local languages and digitally recorded. The project research nurses and their assistants transcribed qualitative and cognitive interviews verbatim and translated the transcripts into English. Translations were peer reviewed by local service colleagues fluent in both English and the relevant local language to check accuracy of translations.

Role of the funding source: The study sponsor (the BIG Lottery Fund UK) had no role in study design; the collection, analysis and interpretation of data; the writing of the report; or the decision to submit the paper for publication.

### Analysis

#### i. Face validity: patients' views (Phases 1a and 1b)

A thematic content analysis of translated transcripts was conducted following import into NVivo v7. The domains of the POS were mapped on to the qualitative interview themes with respect to patient and carer needs, and goodness of fit appraised. Each item was reviewed for appropriateness in light of cognitive interview data, and any training needs to ensure comprehension were noted. Data was presented to the entire project team for discussion and feedback at key points during the analysis process.

#### ii. Construct validity (Phase 2)

POS scores were transformed so that for all items high scores indicated better patient status (i.e. scoring for items 1, 2, 3 and 10 were reversed), in line with MVQoLI scoring. Analyses were undertaken using the weighted subscales, as used in the original validation of the MVQoLI. Before running the correlation data were cleaned, screened for any outliers and distributions of scores checked for normality. Spearman's rank was selected for the correlation analyses as a conservative non-parametric test. Spearman's rank test was used to correlate the POS against the MVQoLI in the following ways: POS total/MVQoLI total score, and POS total for patient items only (1-7)/MVQoLI total score. The MVQoLI symptom subscale was correlated against the sum of POS items 1 (pain) and 2 (symptoms). The MVQoLI interpersonal subscale was correlated against POS item 4 (ability to share feelings). The MVQoLI well being subscale was correlated against the sum of POS items 3 (patient worry), 6 (feeling at peace) and 7 (help and advice). The MVQoLI transcendence subscale was correlated against POS item 5 (life feeling worthwhile). Decisions regarding which POS items to correlate with the MVQoLI subscales were made on the basis of best fit between items in the respective tools.

#### iii. Internal consistency (Phase 3)

Cronbach's alpha was calculated twice, using two datasets from the same sample, collected during assessment of test/re-test reliability.

#### Test/re-test reliability (Phase 3)

Intraclass correlation coefficients (ICC) were calculated for two time points.

#### v. Time to complete (Phase 3)

Median and mean times to complete were calculated from the two POS administrations during the test/re-test reliability phase. Mean, median and ranges of time to complete were also calculated for the MVQoLI, for purposes of comparison.

A level of p < 0.05 (two-tailed) was selected for all tests of significance.

## Results

### Participant characteristics

Validation of the APCA African POS involved interviews with a total of 682 patients and 437 family carers. Respondent characteristics for each validation phase are shown in Table 1. Across the phases of validation, respondents reported 28 different first languages and interviews were conducted in 8 different languages (49.6% IsiZulu, 15.3% English, 12.8% isiXhosa, 6.4% Luganda, 6.3% SeSotho, 5.4% Runyoro, 3.9% Runyankole and 0.4% SeTswana) (N = 720; language data not collected for remaining 399 carers).

**Table 1:** Characteristics of validation study participants (Missing N = 0 unless stated)

	Phase 1a (ii) Face validity: qualitative interview	Phase 1b (ii) Face validity: cognitive interview	Phase 2 (iii) Construct validity	Phase 3 (iv) Internal consistency, (v) Test-retest reliability & (vii) Time to complete
PATIENTS N	90	73^	285	307
Age				
***Mean (SD)***	43.2 (15.4)	44.8 (16.0)	40.1 (12.8)	41.9 (14.1)
**Gender**				
***Female***	58 (64.4%)	49 (67.1%)	197 (69.1%)	207 (67.4%)
**Primary diagnosis**				
***Cancer***	34 (37.8%)	33 (45.2%)	86 (30.2%)	90 (29.3%)
***HIV***	61 (67.8%)	44 (60.3%)	229 (80.4%)	244 (79.5%)
***Of HIV+ pts:***				
**On ART**	39 (63.9%)	24 (54.5%)	127 (55.5%)^a^	140 (57.4%)
**Prior AIDS**				
**diagnosis**	54 (88.5%)	37 (84.1%)	192 (83.8%)	196 (80.3%)
**ECOG Functional status**				
***Fully active***	10 (11.1%)	8 (11.0%)	21 (7.4%)	29 (9.4%)
***Restricted***	24 (26.7%)	24 (32.9%)	65 (22.8%)	79 (25.7%)
***Ambulatory***	17 (18.9%)	17 (23.3%)	79 (27.7%)	71 (23.1%)
***Limited self care***	25 (27.8%)	14 (19.2%)	87 (30.5%)	101 (32.9%)
***Completely disabled***	14 (15.6%)	10 (13.7%)	33 (11.6%)	27 (8.8%)
**Household size**				
***Mean (SD)***	5.2 (5.0)^a^	4.9 (3.1)^a^	5.3 (2.5)^b^	5.5 (3.2)
**Responsible for children?**				
**Yes**	68 (75.6%)	54 (74.0%)	232 (81.4%)	238 (77.5%)
***Mean no. of children (SD)***	2.8 (1.8)	2.9 (1.8)	3.1 (1.96)	3.1 (2.2)
**Location of home**				
***Urban***	23 (25.6%)	23 (31.5%)	53 (18.6%)	79 (25.7%)
***Peri-urban***	26 (28.9%)	26 (35.6%)	53 (18.6%)	52 (16.9%)
***Rural***	41 (45.6%)	24 (32.9%)	179 (62.8%)	176 (57.3%)
**Place of care**				
***Home***	56 (62.2%)	50 (68.5%)	180 (63.2%)	204 (66.4%)
***Inpatient***	27 (30.0%)	16 (21.9%)	74 (26.0%)	79 (25.7%)
***Outpatient***	3 (3.3%)	3 (4.1%)	13 (4.6%)	12 (3.9%)
***Day care***	4 (4.4%)	4 (5.5%)	18 (6.3%)	12 (3.9%)
**Weeks under care**				
***Mean (SD)***	51.4 (85.2)^a^	62.6 (91.2)^a^	46.0 (74.8)	39.1 (69.5)
***Median***	15.0	25.0	12.0	8.0
**FAMILY CARERS N**	38	29	199	200
**Age**			*	*
***Mean (SD)***	44.8 (17.5)^b^	46.9 (18.3)		
**Gender**			*	*
***Female***	32 (84.5%)^b^	26 (89.7%)		
**Household size**			*	*
***Mean (SD)***	7.1 (3.5)^b^	6.8 (3.7)		
**Responsible for children?**			*	*
***Yes***	30 (78.9%)^b^	24 (82.8%)		
***Mean no. of children (SD)***	3.1 (1.6)	3.0 (1.7)		
**Location of home**			*	*
***Urban***	4 (10.5%)	4 (13.8%)		
***Peri-urban***	10 (26.3%)	10 (34.5%)		
***Rural***	21 (55.3%)	15 (51.7%)		
***Missing***	3	0		

^ Subset of sample 1a

* Carer data not collected for Phase 2 and 3.

^a ^Missing = 1

^b ^Missing = 3

### Validity and Reliability

#### i. Face validity: patients' and families' views (Phases 1a and 1b)

Qualitative interviews to map domains of patients and family concern were conducted with a purposive sample of patients (N = 90) and family carers (N = 38). Cognitive interviews were carried out with a subset of these (N = 73 patients, N = 29 carers). Fewer cognitive interviews than qualitative interviews were included in the analysis, as cognitive data from one of the sites was excluded due to deviation from the protocol.

Analysis of in-depth qualitative interviews with patients and carers confirmed that POS items mapped well onto the main themes of identified need: pain and symptoms; treatment; psychological well being; religious belief and spirituality; communication and information; family support and carer needs.

Cognitive interviewing demonstrated good interpretation. The item with most frequent problems in interpretation was question 7: 'Have you had enough help and advice for your family to plan for the future?' for which 13 interviewees gave responses indicative of comprehension difficulties.

During the cognitive interviews, when asked if the POS should include any additional questions. 5 of these gave suggestions for additions, 4 of which related to financial and social support e.g. 'I think if you can ask like "how does a person manage financially?" ‘(Carer, South Africa)’.

#### ii. Construct validity (Phase 2)

In Phase 2 of the validation 285 patients completed the POS and MVQoLI. The Spearman's rank correlations for the POS against MVQoLI items are displayed in Table 2. All correlations are low-moderate, as hypothesized owing to the differences between the constructs of the two measures. The correlation analysis of "best fit" domains between the 2 measures demonstrates that the MVQoLI and APCA African POS are measuring different elements of pain/symptoms and spiritual wellbeing.

**Table 2:** Correlations of MVQoLI against the POS

MVQol Item	POS Item	Correlation coefficient (r)
**MVQoLI total**	POS total*	0.538
**MVQoLI total**	POS total for patient items only*	0.566
**MVQoLI symptom subscale**	Sum (POS Q1* (pain) + Q2* (symptoms))	0.117
**MVQoLI interpersonal subscale**	POS Q4 (sharing feelings)	0.392
**MVQoLI well being subscale**	Sum POS Q6 (peace) + Q3* (worry) + Q7 (help & advice)	0.435
**MVQoLI transcendence subscale**	POS Q5 (life worthwhile)	0.238

* POS items transformed so that for all items high scores indicated better patient status.

#### iii. Internal consistency (Phase 3)

In Phase 3 of the validation, internal consistency was measured on two data sets from the same sample, collected during assessment of test/re-test reliability (N = 307). The α reliability coefficient (Cronbach's Alpha) was 0.60 on both data sets. As hypothesized, this indicates moderate internal consistency.

#### iv. Test/re-test reliability (Phase 3)

The second visit during test-retest reliability was 3-48.5 hours after the first (N = 307). The mean time between visits was 21.2 hours (median 23.2, SD 7.2, range 3-48.45 hours). 44 (14.3%) patients were visited for the second time on the same day as the first visit; 260 (84.7%) were visited the following day, and 2 (0.7%) were visited 2 days later. 1 patient died between the first and second visits and was excluded. 107 family carers were unable to respond to the carer items on both visits and hence were excluded.

High intraclass correlation coefficients (ICC) were found for all items, ranging from 0.78 (symptoms) - 0.89 (total POS score) (see Table 3).

**Table 3:** Intraclass correlation coefficients of scores obtained on first and second visits in test/re-test (patients n = 307)

Item/Total	ICC (single measures)	Confidence interval (95%)	P	Excluded
Q1 pain*	0.785	0.737-0.824	0.001	0.3% (N = 1)
Q2 symptoms*	0.775	0.726-0.816	0.001	0.3% (N = 1)
Q3 worry*	0.824	0.784-0.857	0.001	0.3% (N = 1)
**Q4 able to share**	0.765	0.715-0.808	0.001	0.3% (N = 1)
**Q5 life worthwhile**	0.812	0.770-0.847	0.001	0.3% (N = 1)
**Q6 at peace**	0.777	0.728-0.818	0.001	0.3% (N = 1)
**Q7 help and advice**	0.815	0.773-0.849	0.001	0.3% (N = 1)
**Q8 family info**	0.882	0.847-0.909	0.001	35.2% (N = 108)
**Q9 family confidence**	0.831	0.783-0.870	0.001	35.2% (N = 108)
Q10 family worry*	0.857	0.815-0.890	0.001	35.2% (N = 108)
Total POS score*	0.892	0.859-0.917	0.001	35.2% (N = 108)
Total POS patient items only (Q1-Q7)*	0.876	0.847-0.899	0.001	0.3% (N = 1)

* POS items transformed so that for all items high scores indicated better patient status.

#### v. Time to complete (Phase 3)

The median time to complete the APCA African POS reduced from 7.00 minutes (mean 9.31, SD 6.69) at the first visit, to 5.00 minutes (mean 7.80, SD 7.22) at the second visit. In comparison, the median time to complete for the MVQoLI was 16.00 minutes (mean 19.64, SD 11.76).

Overall levels of missing data were extremely low for both tools across the sample of 285 individuals. Where it did occur, it was of an 'item non-response' type [25], i.e. single items missing. Given the overall low levels of missing data, formal statistical methods to impute missing data were not utilised. Where an item of the MVQoLI subscale was missing, a score was not calculated for that subscale and it was excluded in any analyses (missing data by subscale: global score, symptom, function and well-being subscales, missing n = 0; interpersonal subscale, missing n = 2; transcendent subscale missing n = 9). There were no missing POS scores for items 1-7, which are the items used in the correlation analyses (missing data for items 8-10 are reported in Table 3).

## Discussion

To date, the evidence base in African palliative care has been severely limited by the absence of a locally developed tool for outcome measurement, validated using robust scientific validation methods [8,9]. This study met accepted standards for tool validation [26,27].

The data presented here provide rigorous evidence that the APCA African POS has sound psychometric properties. The tool also appears to have high levels of acceptability and utility in the African clinical setting, which may make it more suitable for use than the MVQoLI. In particular, Namisango et al do not report cognitive interview data from the validation of the MVQoLI in Uganda [13], and the complexity and length of the tool suggest it may be inappropriate for use in many settings in Africa. In the Ugandan study the average time to complete the MVQoLI was between 15 and 35 minutes depending on the performance score [13]; we found a similar mean time of 19 minutes. In contrast, the low mean and median time to complete values for the APCA African POS (mean 8-9 minutes; median 5-7 minutes) indicate that the measure is brief to use and may be easily incorporated into routine clinical assessment.

Those affected by life-limiting disease should have the right to receive evaluated, best quality health care, and appropriate measures are essential to achieving this goal. In settings where resources are limited, resource allocation and provision of care to those with progressive incurable disease should be guided by locally generated and relevant evidence.

As with any tool, we recommend that training and support be provided in its use. This is particularly necessary when a small number of patients described comprehension difficulties on a specific item. The research group is currently developing a manual to provide guidance on applying the tool in clinical audit.

Since its original development, the Palliative Outcome Scale has been developed into different cultural/linguistic versions. There is also a trend in outcome measurement toward the use of core and "add-on" scales (for specific diseases, problems or populations), which may be appropriate in the African setting as the data presented suggest that additional items addressing the socio-economic dimension may be useful.

Application of this tool offers the opportunity to at last address the omissions of palliative care research in Sub-Saharan Africa, to generate local evidence using an appropriate tool, and to incorporate this brief and valid measure into routine clinical audit. The APCA African POS has now been adopted in a number of clinical audit and longitudinal research studies across Africa.

## Conclusions

Despite the strengths of a multi-centre, international approach with early and ongoing input of pan-African clinicians and researchers, and the full validation in diverse inpatient and home care settings, in both HIV and cancer diagnoses, across disease stages and ART use, there are several limitations to our study. Firstly, we have only been able to develop and validate a tool for use in adult populations. We believe that a tool for paediatric palliative use is urgently needed for Africa. Secondly, the translation into many languages is necessary for Africa, and while we have followed best practice, we accept that cultural difference in meaning may potentially lead to different understandings. We have attempted to investigate this through the cognitive interviews. We believe that the value of cognitive interviews in tool validation is that, while all potential comprehension/understanding differences can never be designed out of a tool for wide application, awareness can be applied in training and application. Third, while a version of the MVQoLI has been validated among advanced AIDS patients in Uganda [28], the small number of changes in the newer version and the populations studied in our validation (i.e. different stages of both HIV and cancer across South Africa and Uganda) led us to choose the original version. The lack of a previously validated tool in these populations limited our choices of a comparator. As the MVQoLI was not designed for patients with life limiting illness from the point of diagnosis to the end of life (i.e. the full range of potential palliative care intervention) we plan to investigate how it behaves according to disease stage.

The APCA African POS is currently in use in a number of clinical trials and longitudinal studies across a range of diseases and countries. We believe that use of this tool may significantly advance the measurement, and improvement, of care for African patients and families affected by life-limiting incurable disease.

## Competing interests

The authors declare that they have no competing interests.

## Authors' contributions

There are 12 authors on the submitted manuscript. This study has been very much a collaborative programme of research with full participation from academics and clinicians in our partner African clinical settings. In line with our approach to partnering research in Africa, we have included all colleagues who made a substantive contribution. All authors have read and approved the final manuscript.

RH designed the study, was Principal Investigator, contributed to data analysis and wrote and approved the final draft of the paper. LS managed the study, conducted data analysis and wrote early drafts of the paper. GA collected data for the study and reviewed the paper. ND acted as Principal Investigator at one of the participating sites, managed the research nurse and reviewed the paper. As an African Palliative Care Association representative, JD contributed to project meetings and reviewed the paper. LG was Principal Investigator at one of the participating sites, managed the research nurse and reviewed the paper. TM collected data for the study and reviewed the paper. KM collected data for the study and reviewed the paper. TM served as Principal Investigator at one of the participating sites, managed the research nurse and reviewed the paper. LMS was Principal Investigator at one of the participating sites, managed the research nurse and reviewed the paper. BP was Principal Investigator at one of the participating sites, managed the research nurse and reviewed the paper. IJH helped seek funding for the study, contributed to study design and reviewed the paper.

## Supplementary Material

Additional file 1 **The APCA African POS**. The full and final validated tool. Click here for file
