# Supplementary material for: Validation of a core outcome measure for palliative care in Africa: the APCA African Palliative Outcome Scale
Source: Health Qual Life Outcomes. 2010 Jan 25;8:10. doi: 10.1186/1477-7525-8-10 (PMC2825183; doi:10.1186/1477-7525-8-10)
Supplement: Additional file 1 — The APCA African POS. The full and final validated tool. [file 1477-7525-8-10-S1.DOC]

**Additional File 1:** APCA African POS

|  | **POSSIBLE RESPONSES** | |
| --- | --- | --- |
| ASK THE PATIENT | | |
| **Q1.** Please rate your pain (from 0 = no pain to 5 = worst/overwhelming pain) during the last 3 days | | **0 (no pain)**  **- 5 (worst/overwhelming pain)** |
| **Q2.** Have any other symptoms (e.g. nausea, coughing or constipation) been affecting how you feel in the last 3 days? | | **0 (no, not at all)**  **- 5 (overwhelmingly)** |
| **Q3.** Have you been feeling worried about your illness in the past 3 days? | | **0 (no, not at all)**  **- 5 (overwhelming worry)** |
| **Q4.** Over the past 3 days, have you been able to share how you are feeling with your family or friends? | | **0 (no, not at all)**  **- 5 (yes, I’ve talked freely)** |
| **Q5.** Over the past 3 days, have you felt that life was worthwhile? | | **0 (no, not at all)**  **- 5 (yes, all the time)** |
| **Q6.** Over the past 3 days, have you felt at peace? | | **0 (no, not at all)**  **- 5 (yes, all the time)** |
| **Q7.** Over the past 3 days, have you had enough help and advice for your family to plan for the future? | | **0 (no, not at all)**  **- 5 (as much as wanted)** |
| ASK THE FAMILY CARER | | |
| **Q8.** Over the past 3 days, how much information have you and your family been given? | | **0 (none)**  **- 5 (as much as wanted)**  **N/A** |
| **Q9.** Over the past 3 days, how confident has the family felt caring for the Client? | | **0 (not at all)**  **- 5 (very confident)**  **N/A** |
| **Q10.** Has the family been feeling worried about the Client over the last 3 days? | | **0 (not at all)**  **- 5 (severe worry)**  **N/A** |
